# *In vitro* evaluation of probiotic properties of lactic acid bacteria isolated from the vagina of yak (*Bos grunniens*)

**DOI:** 10.7717/peerj.13177

**Published:** 2022-03-29

**Authors:** Qingli Zhang, Yangyang Pan, Meng Wang, Liang Sun, Yao Xi, Mei Li, Qiaoying Zeng

**Affiliations:** 1 College of Veterinary Medicine, Gansu Agricultural University, Lanzhou, Gansu, China; 2 Technology and Research Center of Gansu Province for Embryonic Engineering of Bovine and Sheep & Goat, Lanzhou, Gansu, China

**Keywords:** Yak, Lactic acid bacteria, Probiotic, Endometritis, Antimicrobial activity, BEECs, Adhesion ability

## Abstract

Bovine endometritis is an inflammatory disease of the uterus that occurs after parturition and can result in the destruction of uterine microecology, disruption of hormone secretion, and even infertility. Problems such as antibiotic residues, pathogen resistance, and microbiota dysbiosis caused by conventional antibiotic therapy cannot be ignored. According to the microecological balance theory, probiotics have the potential to prevent or cure endometritis in cattle. Probiotics can positively influence host physiology by regulating microecological imbalance, modulating immunity, and antagonizing pathogens. Since some probiotics contribute to host health only in their specific natural niches, lactic acid bacteria (LAB) from the vagina may have better potential to fight against vaginal and uterine infection. The yak (*Bos grunniens*) is an ancient and primitive livestock animal that is adapted to high altitude and harsh environments (cold, nutritional deficiencies, and hypoxia). However, to our knowledge, there have been no studies on yak vaginal LAB. Therefore, the purpose of this study was to isolate vaginal LAB from yak, evaluate and compare the probiotic potential and safety of the isolates, and help establish the probiotics library that can be used in the prevention and/or treatment of endometritis. Twenty-five vaginal swabs were collected from healthy yak and cultured in deMan, Rogosa, and Sharpe (MRS) broth. Tentative LAB strains were preliminarily determined through calcium dissolving zone and morphological identification, and the strains were then identified using 16S rRNA gene sequencing. The probiotics of the isolates were detected using cell aggregation, hydrophobicity, resistance to acid and bile salt, adhesion, and antibacterial activities. Additionally, antimicrobial susceptibility, hemolytic activity, and detection of potential virulence factors were determined in order to confirm the safety of these strains. Five isolates were identified: *Leuconostoc mesenteroides*, *Lactobacillus plantarum*, *Enterococcus hirae*, *Lacticaseibacillus camelliae*, and *Lactobacillus mucosae*. All isolates had certain growth resistance, aggregation ability, effective antimicrobial potency against *Escherichia coli*, *Staphylococcus aureus*, and *Salmonella typhimurium*, were sensitive to most antibiotics, and could effectively adhere to bovine endometrial epithelial cells (BEECs). None of the isolates showed hemolytic activity or harbored virulence factors. Our results indicated that the five isolates have considerable potential as probiotics that can be used to prevent and/or treat bovine endometritis. We speculate that a mixture of YD6, YD9, and YD25 may yield better results, although this would require extensive experiments to verify.

## Introduction

Endometritis, inflammation of the uterus caused by bacteria, viruses, fungi, mycoplasma, other infections, or a combination of the above, destroys the microenvironment and disrupts hormone secretion, causing infertility, abortion, postpartum uterine diseases, and, consequently, substantial economic losses to the breeding industry worldwide (Duar et al., 2017; Miranda-CasoLuengo et al., 2019; Zhang et al., 2017a; Sheldon et al., 2009). Traditional treatment of bovine endometritis still relies on antimicrobials (Agrawal et al., 2021). Antimicrobial abuse causes veterinary drug residues and bacterial resistance in the reproductive tract of cows, disrupting the normal microbiota of its mucosa, ultimately leading to the recurrence of endometritis and posing a significant threat to the human food chain (Keijser et al., 2019; Zhang et al., 2017b, Li et al., 2019).

Previous studies have suggested that the uterine microbial composition is highly influenced by vaginal microbes (Molina et al., 2020). Miranda-CasoLuengo et al. (2019) found that the microbiomes of the vagina and uterus were most similar on postpartum day 7. *Lactobacillus* is present in the vagina and uterus of postpartum cows (Gartner et al., 2015). Kudo et al. (2021) found a negative correlation between Trueperella and several bacteria, including *Lactobacillus*, by comparing the abundance of bacterial genera in the uterine microbiota. They suggested that establishing an environment with an increase in bacteria that are generally considered beneficial (such as *Lactobacillus*) may be a possible solution to reduce Trueperella populations and control endometritis (Kudo et al., 2021). According to the microecological balance theory, probiotics have the potential to prevent or cure endometritis in cattle (Yang et al., 2021). Studies have shown that probiotics can positively influence host physiology by regulating microecological imbalance, aiding digestion, modulating immunity, antagonizing pathogens, and resolving diseases (Li et al., 2018; Ng et al., 2009; Van Baarlen, Wells & Kleerebezem, 2013). Numerous lactic acid bacteria (LAB) metabolites, such as organic acids, short-chain fatty acids, hydrogen peroxide, conjugated linoleic acid, bacteriocins, and gamma-aminobutyric acids are related to probiotic biological effects (Upadrasta & Madempudi, 2016; Wang et al., 2018; Ye et al., 2021). Probiotics have the advantages of low toxicity and residue and are pollution-free (Liang et al., 2021), which has piqued the interest of researchers.

Decades ago, the application of LAB in combating uterine diseases was studied (Kummer et al., 1997) and encouraging results were achieved. A recent study found that probiotics could cure endometritis and restore a normal physiological state while avoiding the disadvantages of antimicrobial treatments, such as reductions in the abundance of beneficial microbiota (Yang et al., 2021). One study presented the possibility of applying probiotic preparations and aspartic acid in the treatment of endometritis in dairy cows (Красочко & Снитко, 2020). Genis et al. (2018) found that the vaginal application of the LAB combination could down-regulate the expression of pro-inflammatory cytokines in neutrophils and reduce the infection rate of endometritis.

Of course, the benefits of LAB are comprehensive, complex, and not only limited to the prevention or cure of endometritis. Intravaginal administration of a LAB cocktail could decrease the incidence of clinical endometritis, reduce acute phase protein haptoglobin, and increase milk production (Ametaj et al., 2014). Intravaginal injections of LAB can also accelerate the involution of dairy cow uteruses, lower the incidence of uterine infection, reduce the number of open days, and improve the recovery of the ovarian cycle (Deng et al., 2014, 2015). The presence of *Lactobacillus* in the bovine uterus improved the uterine environment and reproductive performance (Machado et al., 2012; Peter et al., 2018). The culture supernatant of *Lactobacillus acidophilus* had a positive effect on the growth and development of bovine embryos (Li, Brackett & Halper, 2005). During human embryo transfer, colonizing the tip of the transfer catheter with *Lactobacillus crispatus* improved the implantation rate, live, and yield, reduced the infection rate, and increased the pregnancy rate of women undergoing *in vitro* fertilization (Moreno et al., 2016; Sirota, Zarek & Segars, 2014). The number of studies on the impact of LAB on fertility is still limited, so the mechanism is not very clear, but accumulating evidence indicates that vaginal LAB have vast application prospects for preventing and/or treating bovine endometritis and even improving fertility.

As ancient and primitive livestock, yak can make full use of scarce natural resources in high-altitude areas and have strong adaptability to harsh environmental conditions such as hypoxia, low temperature, low pressure, and short grasses. Their ability to resist disease and stress is potentially related to the probiotics in their bodies. A previous study determined that *Bacillus subtilis* and *Bacillus velezensis* isolated from Tibetan yaks increased the overall growth performance and ameliorated the blood parameters related to inflammation and immunity in mice (Li et al., 2019). *Lactobacillus johnsonii* and *Lactobacillus mucosae* isolated from fecal samples of Tibetan yaks lowered the rates of diarrhea and mortality of mice (Wang et al., 2018). *Lactobacillus plantarum* isolated from Tibetan yak milk fed to rats on a high cholesterol diet promoted notable declines in serum, liver cholesterol, triglyceride levels, and lipid deposition in the cytoplasm of the rat’s liver tissue (Ding et al., 2017). The evolution of LAB means that today’s *Lactobacillus* species have undergone great changes in their dependence on environmental niches and host specificity. Since certain species contribute to host health only in their specific natural niches (Duar et al., 2017), researchers are trying to screen probiotic bacteria from their native niches. Additionally, probiotics isolated from cattle vaginal samples have been examined for their properties in order to prevent or treat uterine infections (Deng, 2015). Research has found that vaginal LAB could regulate the vaginal ecosystem mainly through cell surface adhesion, colonization, space occupation, the inhibition of pathogens, and immunostimulation (Aroutcheva et al., 2001). Although several strains of LAB isolated from cow vaginal mucus have been reported, there have been no studies on yak vaginal probiotics. Therefore, in this study we explored the probiotic potential and safety of vaginal LAB from yak *via* screening and *in vitro* tests in order to help establish a probiotics library to be used in the prevention and/or treatment of endometritis.

## Materials and Methods

### Bacterial strains, cells, and culture conditions

Standard bacterial strains of *Escherichia coli* (*E. coli* ATCC (American Type Culture Collection) 25922), *Staphylococcus aureus* (*S. aureus* ATCC 25923), and *Salmonella typhimurium* (*Salm. typhimurium* CMCC (National Center for Medical Culture Collections) 50115) were purchased from Beijing Biobw Biotechnology Co., Ltd. (Beijing, China) to determine cell aggregation and antibacterial activity. They were aerobically cultured in Luria Bertani (LB) broth, Mueller Hinton (MH) broth, or MH agar plates for 24 h at 37 °C. Bovine endometrial epithelial cells (BEECs) were gifted by Xiangguo Wang (Beijing University of Agriculture) and cultured at 37 °C in 5% CO_2_ in Dulbecco’s Modified Eagle Medium: F-12 (DMEM/F12) (Hyclone, Logan, UT, USA) supplemented with 10% FBS (Pansera, Aidenbach, Germany).

### Bacterial isolation from yak vagina

Vaginal secretions from healthy yak were collected with sterile cotton swabs at the Linxia Abattoir in Gansu Province, China. They were stored in normal saline and transported back to the laboratory at 4 °C. The samples were cultured in MRS medium for 12 h, spotted onto MRS agar supplemented with 2% (w/v) CaCO_3_, and anaerobically incubated at 37 °C for 48 h. A clear zone around the colony was formed after lactic acid reacted with CaCO_3_. As previously described, the milky white convex colonies with transparent circles were selected and incubated as tentative LAB colonies (Chang, Lee & Chang, 2007). Following Gram staining, we observed the cell morphology using optical microscopy (Zeiss, Oberkochen, Germany) with a 100× oil immersion lens. The tentative LAB colonies were purified by subculture and stored at –80 °C with 25% (v/v) glycerol.

### Molecular identification

The tentative LAB were cultured in MRS broth overnight. According to standard protocols, total genomic DNA was extracted from 2 mL of fresh culture medium using a bacterial genomic DNA extraction kit (TIANGEN, Beijing, China). Biochemical identification was conducted by sequencing 16S rRNA gene using universal primers (27F 5′-AGAGTTTGATCCTGGCTCAG-3′; 1492R, 5′-GGTTACCTTG TTACGACTT-3′) and the previously described polymerase chain reaction (PCR) reaction conditions (Vaz-Moreira et al., 2009; Alegria et al., 2009).

The PCR amplifications were performed in a final volume of 25 µL containing 12.5 µL Taq master mix (2×) DNA polymerase (Vazyme, Nanjing, China), 1 µL of DNA template (50 ng DNA), 1 μL (10 μM) of each primer, and 9.5 µL H_2_O. The amplification reaction was carried out in an Eppendorf Mastercycle thermocycler under the following conditions: 5 min at 94 °C, 35 cycles of 30 s at 94 °C, 30 s at 55 °C, and 90 s at 72 °C, and a final elongation at 10 min at 72 °C. PCR products were size-verified in 1% (w/v) agarose (Genewiz, Suzhou, China), and the sequences were compared with the 16S rRNA sequence in the National Center for Biotechnology Information (NCBI) public database using the BLAST (Basic Local Alignment Search Tool) program. Phylogenetic analysis was conducted using MEGA 7.0 software with the distance option of the Kimura two-parameter model and clusters with neighbor connection (NJ) method based on bootstrap values of 1,000 (Kumar, Stecher & Tamura, 2016).

### Cell aggregation

Autoaggregation (aggregation between microorganisms of the same strain) properties were performed based on previously described methods with minor modifications (Li et al., 2020; Wang et al., 2021). Briefly, the LAB strains were cultured in MRS broth at 37 °C for 18 h. The cultures were harvested (5,000×*g*, 10 min, 4 °C), washed three times with PBS (pH 7.0), and resuspended in PBS. The bacterial solution was incubated (aerobic and static) at 37 °C, and after 2 h the optical density (OD600_nm_) of the bacterial supernatant was measured. Autoaggregation (%) was calculated as:

$\rm{Autoaggregation \% = [(A_0-A_t)/A_t] \times 100}$

where A_0_ represented the absorbance at time *t* = 0 h, and A_t_ represented the absorbance taken at 2 h.

The bacterial suspension used for coaggregation (aggregation between genetically different strains) was prepared as above. We mixed 2 mL suspension of LAB strains with 2 mL suspension of three indicator strains (*S. aureus* ATCC 25923, *E. coli* ATCC 25923, and *Salm. typhimurium* CMCC 55522) respectively, then incubated them at 37 °C without agitation. After 2 h, the absorbance of the mixture was determined. Coaggregation (%) was calculated as follows:

$\rm{Coaggregation \% = [(A_p+A_i) - 2A_{mix}/(A_p+A_i)] \times 100}$

where A_p_ and A_i_ represented the OD600_nm_ of the three standard bacterial strains and five isolates before mixing, and A_mix_ denoted the OD600_nm_ of the mixed bacterial supernatant at 2 h (Wang et al., 2021).

### Hydrophobicity

To assess the degree of surface hydrophobicity, the microbial adhesion to hydrocarbon (MATH) method was adopted with minor modifications (Rosenberg, Judes & Weiss, 1983; Slizewska, Chlebicz-Wojcik & Nowak, 2021). LAB strains were washed twice in PBS, harvested by centrifugation (5,000×*g*, 10 min, 4 °C), and re-suspended in PBS. We measured the optical density of bacterial suspension using spectrophotometry (Biochrom GeneQuant, Cambridge, UK) and adjusted the OD600_nm_ within the range of 1.0 ± 0.1. Each bacterial suspension (3 mL) was mixed with xylene, ethyl acetate, and n-hexadecane (1 mL, Macklin, Shanghai, China), vortexed for 2 min for emulsion formation, and incubated at 37 °C for 1 h. We then measured the OD600_nm_ of the aqueous layer. The hydrophobicity was calculated using the following equation:

$\rm{Hydrophobicity \% =[(A_0 - A_t)/A_0] \times 100}$

where A_0_ represented the absorbance at time *t* = 0 h, and A_t_ represented the absorbance taken at 1 h (Rastogi, Mittal & Singh, 2020).

### Resistance to acid and bile salt

An improved method was used to measure the strain viability in acid and bile salt (Kobierecka et al., 2017). The test solutions included MRS broth adjusted to pH 3.0 with 1 M HCl, MRS broth containing 0.3% bile salt, and the control MRS broth (Li et al., 2020). Briefly, 1 mL of bacterial solution (1 × 10^8^ cfu/mL) was added into 7 mL of the three detection solutions separately, repeated 3 times, and incubated at 37 °C for 3 h. Next, 100 µL of the culture was serially diluted, plated on MRS agar, and incubated at 37 °C for 48 h. The viable number of cells was expressed in the form of log cfu mL^–1^.

### Adhesion

An adhesion assay was conducted using a previously described method with slight modifications (Dowdell et al., 2020). Briefly, the BEECs were incubated in DMEM/F12 with 10% FBS in a 24-well plate to form confluent monolayer cells. The LAB strains were grown in MRS at 37 °C for 24 h, centrifuged, washed with PBS, and the strain density was adjusted to 10^8^ CFU/mL in DMEM/F12 medium. First, we rinsed the BEECs monolayer twice with PBS, added 2 mL of DMEM/F12 serum-free medium and 100 μL of bacterial suspension, and incubated it at 37 °C in 5% CO_2_ for 2 h. Then, we discarded the culture medium and carefully washed the wells twice with PBS to remove non-adherent bacteria. Each well was lysed with 100 µL PBS containing 0.1 µL Triton X-100 at 37 °C for 15 min. The lysate containing adherent bacteria was diluted and plated on MRS plates in triplicate and incubated at 37 °C. After 48 h, the number of viable bacteria was counted (Dowdell et al., 2020).

### Antibacterial activities

Inhibiting the growth of pathogens is one of the most important functions of probiotics (Sahoo et al., 2015). In this experiment, the agar diffusion method was used to evaluate the antibacterial activity of LAB against the indicator bacteria. *E. coli* ATCC 25922, *S. aureus* ATCC 25923, and *Salm. typhimurium* CMCC 50115 were used as the indicator pathogens. The three pathogens were cultured in LB broth at 37 °C for 12 h, and the concentration of the bacterial solution was adjusted to 10^7^ CFU/mL with LB. The isolates were cultured in MRS broth at 37 °C for 24 h, and cell-free culture supernatants (CFCS) were prepared by centrifugation (10,000×*g*, 10 min, 4 °C). First, we spread 100 µL of three pathogens evenly on the MH plate. After the surface was dry, we placed the Oxford cup evenly on the MH plate, added 100 µL CFCS to the Oxford cup, and incubated at 37 °C for 24 h (Kobierecka et al., 2017). MRS broth medium (100 µL) was used as a negative control. The diameter of the transparent zones was scored as follows: less than or equal to 9 mm (negative, –), 9–12 mm (weak, +), 12–16 mm (strong, ++), and more than 16 mm (very strong, +++).

### Safety assessment

#### Antimicrobial susceptibility

The isolates were tested for antimicrobial susceptibility to 12 antimicrobials (ceftriaxone (30 μg), ciprofloxacin (5 μg), ampicillin (10 μg), rifampicin (5 μg), kanamycin (30 μg), streptomycin (10 μg), tetracycline (30 μg), gentamicin (10 μg), chloramphenicoll (30 μg), erythromycin (15 μg), clindamycin (2 μg), and cephalothiophene (30 μg)) using the disk diffusion method. All antimicrobial paper disks were purchased from Hangzhou Binhe Microorganism Reagent Co. (Hangzhou, China). First, we aspirated 100 µL of the isolates (1 × 10^8^ CFU/mL) onto the MRS agar plate and spread it evenly, placed the antimicrobial paper disks on the plates at equal intervals, incubated the plates at 37 °C for 24 h, and measured the diameter of the inhibition zone with a vernier caliper. According to the Clinical and Laboratory Standards Institute (CLSI) 2012, the zone diameters were classified as resistant (R), intermediate (I), or sensible (S). Since CLSI did not have criteria for LAB, this trial referred to published articles for breakthrough points and antimicrobial doses: gentamycin (R ≤ 6 mm; I: 7–9 mm; S ≥ 10 mm), erythromycin (R ≤ 13 mm; I: 13–23 mm; S ≥ 23 mm) (Haghshenas et al., 2015), streptomycin (R ≤ 11 mm; I: 12–14 mm; S ≥ 15 mm) (Liasi et al., 2009), and other antimicrobials (R ≤ 15 mm; I: 15–20 mm; S ≥ 21 mm) (Halder et al., 2017).

#### Hemolytic activity

The isolates and *S. aureus* were cultured overnight in MRS and LB broth, respectively, and the bacterial liquid was streaked on the defibrinated sheep blood plates. After incubating at 37 °C for 24–48 h, the plates were screened for α-haemolysis (grey-green halo), β-haemolysis (transparent halo), or γ-hemolysis (no zone effect) results. *S. aureus* was examined for clear zones around the colonies (β-hemolysis) and was used as positive control (Moreno et al., 2018).

#### Detection of potential virulence factors

We used previously reported primers and conditions to detect the presence of eight virulence genes (*Ace* (collagen-binding protein), *Agg* (aggregation substance), *Asa1* (Aggregation substance gene), *Cpd* (sex pheromone peptides), *CylA* (cytolysin), *ClyB* (cytolysin), *EfaAfs* (cell wall adhesins) and *GelE* (gelatinase)) using PCR amplification (Al-Talib et al., 2015; Eaton & Gasson, 2001; Espeche et al., 2012; Vankerckhoven et al., 2004). The PCR primers, reaction conditions, amplified fragment sizes, and references are summarized in Table 1. PCR amplifications were performed in a 25 µL reaction volume, typically containing 12.5 µL Taq master mix (2×) DNA polymerase (Vazyme, Nanjing, China), 1 µL of DNA template (50 ng DNA), 1 μL (10 μM) of each primer, and 9.5 µL H_2_O. The cycle profile was as follows: initial denaturation (94 °C for 5 min), followed by 35 cycles of denaturation (94 °C for 30 s), annealing at an appropriate temperature (Table 1) for 30 s and elongation (72 °C for 30–75 s), completed by a final elongation (72 °C for 10 min). PCR products were analyzed in 1% (w/v) agarose, stained with GoldView I nuclear staining dyes (Solarbio, Beijing, China), and visualized under UV transilluminator.

**Table 1:** PCR primers, annealing temperatures, and amplicon size used to detect the putative virulence genes in the isolates.

Primers	Sequences (5’–3’)	Tm (°C)	Amplicon size (bp)	References
Ace	Ace-F: CAGGCCAACATCAAGCAACA	65	125	Al-Talib et al. (2015)
	Ace-R: GCTTGCCTCGCCTTCTACAA			
Agg	Agg-F: AAGAAAAAGAAGTAGACCAAC	53	1553	Espeche et al. (2012)
	Agg-R: AAACGGCAAGACAAGTAAATA			
Asa1	Asa1-F: GCACGCTATTACGAACTATGA	56	375	Vankerckhoven et al. (2004)
	Asa-R: TAAGAAAGAACATCACCACGA			
Cpd	Cpd-F: TGGTGGGTTATTTTTCAATTC	50	782	Eaton & Gasson (2001)
	Cpd-R: TACGGCTCTGGCTTACTA			
CylA	CylA-F: ACTCGGGGATTGATAGGC	60	688	Vankerckhoven et al. (2004)
	CylA-R: GCTGCTAAAGCTGCGCTT			
ClyB	ClyB-F: ATTCCTACCTATGTTCTGTTA	56	843	Vankerckhoven et al. (2004)
	ClyB-R: AATAAACTCTTCTTTTCCAAC			
EfaAfs	EfaAfs-F: GACAGACCCTCACGAATA	56	705	Eaton & Gasson (2001)
	EfaAfs-R: AGTTCATCATGCTGTAGTA			
GelE	GelE-F: CGAAGTTGGAAAAGGAGGC	50	372	Al-Talib et al. (2015)
	GelE-R: GGTGAAGAAGTTACTCTGA			

### Statistical analysis

The phylogenic tree was performed using MEGA 7 software and the figures were drawn using GraphPad Prism 8.0. Date were presented as mean ± SD from at least three independent experiments. The groups were compared and analyzed *via* SPSS 26.0 software and one-way ANOVA, and followed the least significant difference (LSD) and Duncan’s analysis for *post hoc* multiple comparisons of treatment means. Statistical significance was defined at *P* < 0.05.

## Results

### Isolation of LAB

**Figure 1:**
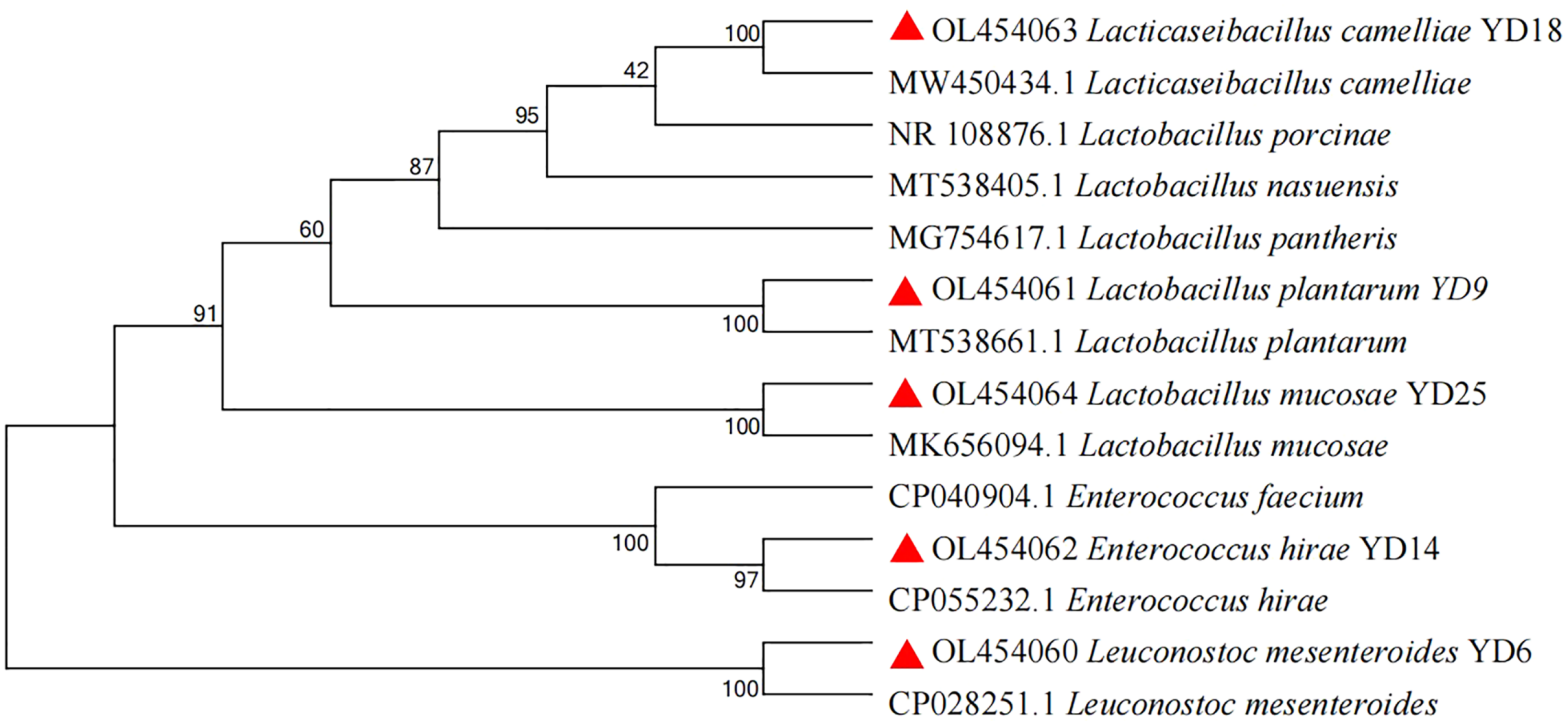
Phylogenetic tree of five isolates based on the neighbor-joining distance analysis of 16S rRNA gene sequences.

In this experiment, 25 presumptive LAB were recovered from the isolation plates. Five presumptive LAB (YD6, YD9, YD14, YD18, and YD25) were selected by comparing the calcium soluble circle size and colony morphology after purification and incubation. The Gram-staining and cell morphology results showed that YD6 and YD14 were Gram-positive cocci, and YD9, YD18, and YD25 were Gram-positive bacilli. Comparative 16S rRNA gene sequence analysis revealed that isolates YD6, YD9, YD14, YD18, and YD25 had high homology (99–100%) with *Leuconostoc mesenteroides* (*Leuc. mesenteroides*), *Lactobacillus plantarum* (*L. plantarum*), *Enterococcus hirae* (*E. hirae*), *Lacticaseibacillus camelliae* (*L. camelliae*), and *Lactobacillus mucosae* (*L. mucosae*), respectively (Figs. S1 and S2). The phylogenetic tree (Fig. 1) was constructed using MEGA 7.0 with the neighbor-joining algorithm and a Kimura two-parameter model. The Genbank accession numbers of each isolate and selected strains used in the phylogenetic studies are presented in Fig. S3.

### Cell aggregation

Table 2 represents the percentage of autoaggregation and coaggregation with three pathogenic bacteria of five LAB isolates. The strains exhibited diverse autoaggregation (17.03–30.12%) and coaggregation abilities (5.36–20.32%) for three pathogens. YD6, YD9, and YD18 exhibited high coaggregation ability (18.08%, 16.65%, and 16.99%) with *E. coli*, and YD9 possessed the highest autoaggregation. YD6 had high coaggregation ability (15.18%) with *S. aureus*, and YD6 showed high coaggregation ability (20.32%) with *Salm. typhimurium*.

**Table 2:** The percentage of autoaggregation and coaggregation with *E. coli*, *S. aureus*, and *Salm. typhimurium* by five isolates.

Isolates	Autoaggregation		Coaggregation	
		*E. coli*	*S. aureus*	*Salm. typhimurium*
YD6 YD9 YD14 YD18 YD25	21.24 ± 1.02 d 30.12 ± 0.49 a 24.85 ± 0.80 c 26.49 ± 0.68 b 17.03 ± 0.59 e	18.08 ± 0.94 a 16.65 ± 1.26 a 10.12 ± 1.66 b 16.99 ± 1.18 a 11.48 ± 1.81 b	15.18 ± 0.35 a 5.36 ± 0.41 c 8.19 ± 0.50 b 5.45 ± 0.57 c 6.13 ± 0.79 c	20.32 ± 1.87 a 16.56 ± 0.74 b 14.83 ± 1.98 b 16.83 ± 1.45 b 10.78 ± 1.46 c

**Note:**

Values expressed as mean ± SD. Different letters represent significant difference, *P* < 0.05.

### Hydrophobicity

**Figure 2:**
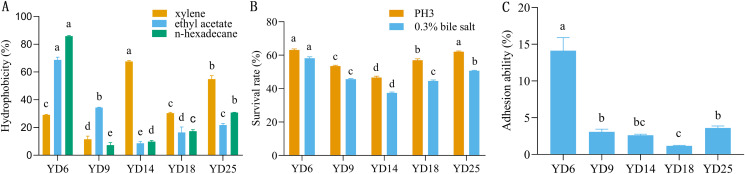
The hydrophobicity, acid and bile salt resistance, and adhesion ability of LAB isolates. (A) Hydrophobicity percentages of LAB isolates to xylene, ethylacetate and n-hexadecane. (B) The acid and bile salt tolerance of LAB isolates. (C) Percent adhesion values of LAB to BEECs. Values expressed as mean ± SD. Different letters represent significant difference, *P* < 0.05.

The five LAB isolates showed varied cell surface hydrophobicity to three organic reagents (Fig. 2A). For xylene, ethyl acetate, and n-hexadecane, the percentages of hydrophobicity ranged from 11.53% to 67.61%, 8.60% to 68.73%, and 7.32% to 85.84%, respectively. YD14 exhibited the strongest adsorption capacity to xylene (67.61%) and YD6 possessed the maximum affinity toward ethyl acetate (68.73%) and n-hexadecane (85.84%).

### Resistance to acid and bile salt

The acid and bile salt tolerance results for the five isolates are provided in Fig. 2B. The five isolates had different resistance to acids and bile salts. Among these strains, YD6 (63.09%) and YD25 (62.07%) showed higher acid resistance than YD18 (56.99%), YD9 (53.49%), and YD14 (46.64%). After exposure to bile salts for 3 h, YD6 (58.15%) exhibited the greatest bile resistance, followed by YD25 (50.68%), YD9 (45.66%), YD18 (44.59%), and YD14 (37.46%).

### Adhesion

The five strains exhibited diverse adhesion abilities to cells, ranging from 1.18% to 14.1% (Fig. 2C). YD6 exhibited the greatest ability to adhere to BEECs (14.1%), followed by YD25 (3.61%), YD9 (3.07%), YD14 (2.63%), and YD18 (1.18%).

### Antibacterial activities

Table 3 shows the results of the antibacterial activity of five strains against three pathogens. YD9 had the strongest antimicrobial activity against *E. coli* (16.36 mm) and *Salm. typhimurium* (17.86 mm), and YD25 exhibited the most potent antimicrobial activity against *S. aureus* (14.96 mm).

**Table 3:** The inhibition zone diameters (mm) of five strains against *E. coli*, *S. aureus*, and *Salm. typhimurium*.

Isolates	Indicator pathogens		
	*E. coli* ATCC 25922	*S. aureus* ATCC 25923	*Salm. typhimurium* CMCC 50115
YD6	13.63 ± 0.57 ++ b	13.50 ± 0.50 ++ bc	14.13 ± 0.32 ++ c
YD9	16.36 ± 0.55 +++ a	13.00 ± 0.30 ++ c	17.86 ± 0.50 +++ a
YD14	12.33 ± 0.57 ++ c	14.50 ± 0.55 ++ ab	14.33 ± 0.57 ++ c
YD18	15.50 ± 0.51 ++ a	12.83 ± 0.28 ++ c	16.23 ± 0.55 +++ b
YD25	15.93 ± 0.30 ++ a	14.96 ± 0.89 ++ a	17.10 ± 0.30 +++ ab

**Note:**

Values expressed as mean ± SD. Different letters represent significant difference, *P* < 0.05.

### Safety assessment

#### Antimicrobial susceptibility

**Figure 3:**
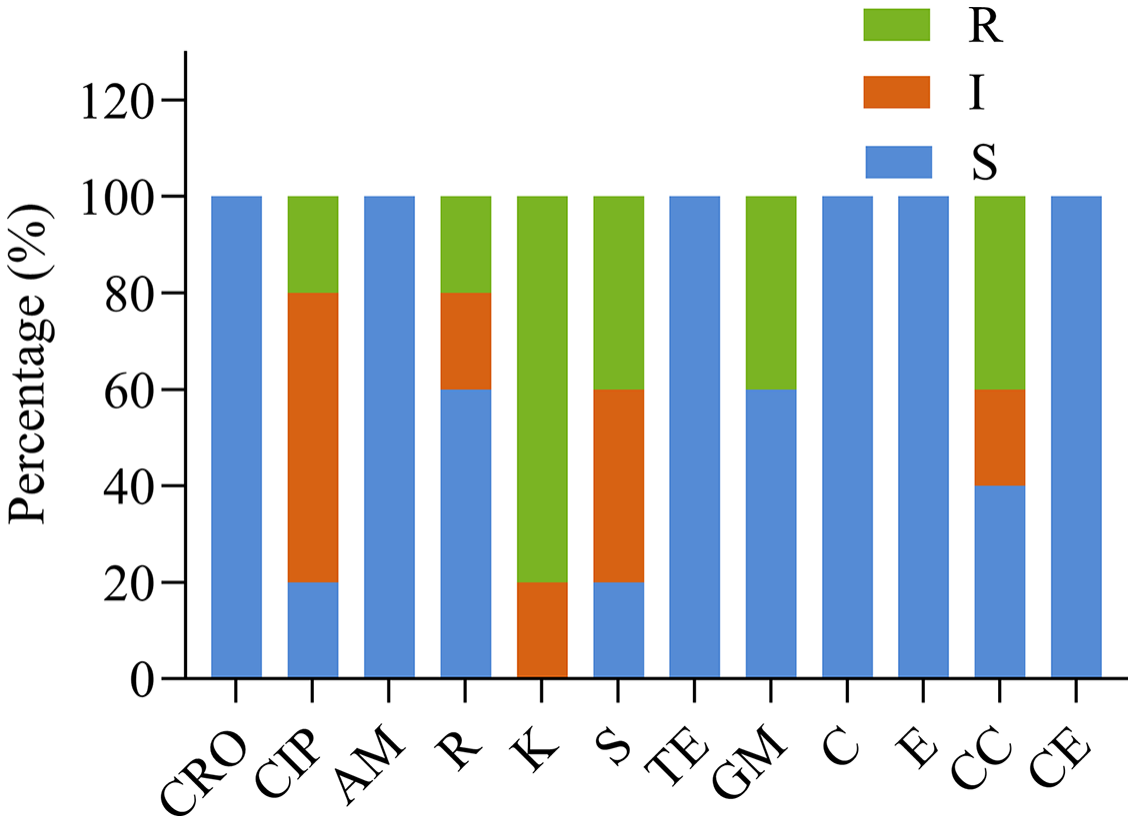
The antibiotic resistance of the LAB isolates against 12 tested antibiotics. CRO, Ceftriaxone; CIP, ciprofloxacin; AM, ampicillin; R, rifampicin; K, kanamycin; S, streptomycin; TE, tetracycline; GM, gentamicin; C, chloramphenicol; E, erythromycin; CC, clindamycin; CE, cephalothiophene. The total number of LAB strains was taken as 100%. S, sensitive; I, intermediately resistant; R, resistant.

Figure 3 and Table 4 show the susceptibility of the five isolates to 12 different antimicrobials. All strains were susceptible to ceftriaxone, ampicillin, tetracycline, chloramphenicol, and erythromycin, and each of the five strains was resistant to at least one antimicrobial. Four of five *Lactobacillus* strains showed high resistance to kanamycin. The impact of other antimicrobials against isolates ranged from susceptibility to resistance. YD6 was intermediately resistant to kanamycin, and resistant to ciprofloxacin and clindamycin. YD9 was intermediately resistant to ciprofloxacin and cephalothiophene, and resistant to kanamycin, streptomycin, gentamicin, and clindamycin. YD14 was susceptible to all of the antimicrobials, except for kanamycin. YD18 was intermediately resistant to clindamycin, and resistant to kanamycin, streptomycin, and gentamicin. YD25 was intermediately resistant to ciprofloxacin, and streptomycin, and resistant to rifampicin and kanamycin.

**Table 4:** Antibiotic susceptibility of selected LAB strains.

Strains	Antibiotic susceptibility zone of inhibition in mm											
	**CRO**	**CIP**	**AM**	**R**	**K**	**S**	**TE**	**GM**	**C**	**E**	**CC**	**CE**
YD6	S	R	S	S	I	S	S	S	S	S	R	S
YD9	S	I	S	S	R	R	S	R	S	S	R	S
YD14	S	I	S	I	R	I	S	S	S	S	S	S
YD18	S	S	S	S	R	R	S	R	S	S	I	S
YD25	S	I	S	R	R	I	S	S	S	S	S	S

**Notes:**

CRO, Ceftriaxone; CIP, ciprofloxacin; AM, ampicillin; R, rifampicin; K, kanamycin; S, streptomycin; TE, tetracycline; GM, gentamicin; C, chloramphenicol; E, erythromycin; CC, clindamycin; CE, cephalothiophene. Erythromycin results based on R ≤ 13 mm; I: 13–23 mm; S ≥ 23 mm. Gentamycin results based on R ≤ 6 mm; I: 7–9 mm; S ≥ 10 mm. Streptomycin results based on R ≤ 11 mm; I: 12–14 mm; S ≥ 15 mm. S, susceptible (zone diameter, ≥21); I, intermediate (zone diameter, 15–20 mm); R, resistant (zone diameter, ≤15 mm).

#### Hemolytic activity and detection of potential virulence factors

*S. aureus* produced clear zones of hemolysis around the colonies (β-hemolysis). However, the five isolates showed no zone effect (γ-hemolysis) on red blood cells, which indicated non-hemolytic activity (Fig. S3). YD6 and YD14 had no virulence genes (Fig. S4).

## Discussion

As the demand for animal products increases, the use of antimicrobials in livestock and poultry breeding has also received more and more attention. Researchers committed to studying safe and non-toxic alternatives to antimicrobials are interested in the application of probiotics in promoting health and improving livestock production performance. Currently, LAB is being used as a probiotic to treat various diseases and as a feed additive to promote growth and enhance immunity.

Yaks are able to live and reproduce freely at high altitudes, which may be related to their internal microbiota. However, there is limited information on yak probiotics, especially their vaginal probiotics. LAB are essential species of a healthy vaginal microbiota and play an irreplaceable role in a healthy reproductive system. The purpose of this study was to screen and select LAB with beneficial properties that can be potentially used as microecological agents or biological feed additives for preventing or treating bovine endometritis.

To our knowledge, this is the first study exploring the probiotic properties of five vaginal LAB (*Leuc. Mesenteroides*, *L. plantarum*, *E. hirae*, *L. camelliae*, and *L. mucosae*) isolated from yak. Although previous studies examined *L. mucosae* isolated from the intestines and faeces from a variety of animals, this study investigated *L. mucosae* and *L. camelliae* isolated from yak vagina. The LAB species in yak vagina were different from the previously reported types of LAB found in cow vagina, which may be due to different host species, environments, or culture conditions. LAB widely exist in cow feces, fence soil, feed rations, and other external environments. We speculate that they may eventually settle in the vagina through eating or mating, although the source of LAB in yak vagina is unclear. Potential probiotics must be systematically identified, characterized, and tested for their effectiveness and safety in related applications. Therefore, we conducted a series of *in vitro* probiotic evaluations and safety tests on five isolates.

Autoaggregation (aggregation between microorganisms of the same strain) and coaggregation (aggregation between genetically different strains) are related to the ability of bacteria to form biofilms and adhere to host epithelial cells or mucosa (Del Re et al., 2000). LAB’s autoaggregation ability is related to the proteins in the culture supernatant and the proteins or lipoproteins on the cell surface. LAB coaggregation with pathogens can form a barrier that prevents pathogens from colonizing, thus exerting the characteristics of probiotics (Collado, Meriluoto & Salminen, 2007). Our experimental results showed that YD9 exhibited the strongest autoaggregation ability among the five strains, YD6 showed the strongest coaggregation ability with three pathogens, and YD9 and YD18 possessed high coaggregation ability to *E. coli*. Ultimately, we concluded that YD9 has outstanding cell aggregation abilities.

Hydrophobic and adhesive properties are connected to the composition of the bacterial membrane. If bacteria have strong self-aggregation and strong hydrophobicity, they are likely to also have strong adhesion to cells (Del Re et al., 2000). Due to differences in cell physiological state or medium composition, the hydrophobicity/hydrophilicity and surface charge of bacteria differ between different strains. In this experiment, YD6 showed the strongest hydrophobicity to both ethyl acetate and n-hexadecane, while YD14 exhibited the strongest hydrophobicity against xylene.

Stability at a low pH and high bile salt in the stomach and digestive tract is an essential characteristic of oral probiotics (Khalil et al., 2018). Acid and bile salt resistance are therefore an important criterion for selecting probiotic LAB, and are some of the main factors affecting the survival probability of LAB. Currently, there are two methods used to detect the number of bacteria resistant to acid and bile salt. The first measures the OD600 value, and the other counts the number of viable cells through the pouring plate. We used the latter in this experiment because this method can better reflect the number of live bacteria. In this study, YD6 and YD25 showed high acid resistance, and YD6 possessed high bile salt resistance.

Bacterial adhesion to tissue is considered the first and key step of microbial colonization (Luna-Guevara et al., 2019). Probiotics can compete with harmful bacteria for both indispensable nutrients and niches (Deng, 2015). The adhesion of LAB to epithelial and mucosal surfaces is a very complex process that involves many different factors (Miljkovic et al., 2016). The adhesion results indicated that YD6 had the strongest ability to adhere to BEECs (14.1%), followed by YD25 (3.61%), YD9 (3.07%), YD14 (2.63%), and YD18 (1.18%). Bacteria’s hydrophobicity is the main factor that determines the non-specific adhesion of bacteria on the surface of the host cell (Zeng et al., 2021). The hydrophobicity of the bacterial cell surface is usually used to predict adhesion. The hydrophobicity results showed that YD14 exhibited the strongest adsorption capacity to xylene (67.61%), and YD6 possessed the maximum affinity toward ethyl acetate (68.73%) and n-hexadecane (85.84%). We concluded that YD6 exhibited the highest hydrophobicity and highest adhesion to BEECs. This result showed that the hydrophobicity of bacteria was positively correlated with its adhesion, which is consistent with previous studies.

Another essential feature of *in vitro* vaginal probiotic strain selection is the inhibition of pathogenic microorganisms (Rodriguez et al., 2011). LAB can produce large amounts of antimicrobial metabolites that can inhibit the growth of various pathogenic bacteria, such as hydrogen peroxide, diacetyl, lactic acid, organic acids, and bacteriocin. The results showed that the five isolates were able to inhibit *E. coli*, *S. aureus*, *and Salm. Typhimurium*. YD9, YD18, and YD25 possessed strong antimicrobial activity against *E. coli*; YD9 showed the strongest antimicrobial activity against *Salm. Typhimurium*; and YD25 exhibited the strongest antimicrobial activity against *S. aureus*.

Probiotic LAB strains must be susceptible to most currently available antimicrobials. In this study, all LAB isolates showed susceptivity to broad-spectrum antimicrobials. Isolating probiotics with low drug resistance from yak vagina may be influenced by the fact that the yak is free-ranged and rarely treated with antimicrobials. Most of the *Lactobacillus* genus is resistant to aminoglycosides such as gentamycin and streptomycin (El-Deeb et al., 2020). None of the isolates showed resistance to gentamycin, and only YD9 and YD18 were resistant to streptomycin, which is an aminoglycoside drug. Due to the lack of cytochrome-mediated drug transport in LAB, aminoglycoside resistance is considered an inherent feature of LAB and other anaerobic bacteria (Sirichoat et al., 2020).

Although probiotics have been used as food-grade microorganisms for a long time, *Enterococci* are controversial because some species harbor a series of virulence factors that are associated with many human and animal infections. Therefore, the potential toxicity-related factors of *Enterococci* must be considered and evaluated (Foulquie Moreno et al., 2006). In this study, none of the isolates caused sheep red blood cell lysis or harbored virulence factors, indicating that they can be used safely as candidate strains for probiotics.

Finally, we conducted a comparative analysis of the experimental results and found that YD6 had the best performance on coaggregation pathogens, hydrophobicity, and adherence to BEECs; YD9 possessed high self-aggregation and the strongest antimicrobial activity against *E. coli* and *Salm. Typhimurium*; and YD25 exhibited the most potent antimicrobial activity against *S. aureus*. However, none of the strains excelled in all aspects. Other experimental studies have provided useful and encouraging results for the use of LAB combinations in the prevention and treatment of uterine and vaginal infectious diseases. We speculate that the mixed preparation of YD6, YD9, and YD25 may be effective in the prevention and/or treatment of bovine endometritis. This would require extensive experiments to verify and more *in vivo* safety and effectiveness evaluations to determine its practical application.

## Conclusion

This is the first study to evaluate the probiotic characteristics of LAB isolated from the vagina of yaks. The five strains were *Leuc. Mesenteroides*, *L. plantarum*, *E. hirae*, *L. camelliae*, and *L. mucosae*. A series of previous safety (antimicrobial susceptibility, hemolytic activity, and detection of potential virulence factors), and *in vitro* physiological function tests (cell aggregation, hydrophobicity, resistance to acid and bile salt, adhesion, and antibacterial activities) showed the potential probiotics of the five isolates. The five strains were sensitive to most antimicrobials without hemolytic activity or virulence factors, suggesting they have considerable potential as probiotics used to prevent and/or treat bovine endometritis. However, no strain excelled in every aspect (YD6 had the best performance in aggregation, hydrophobicity, and adherence to BEECs; YD9 possessed the strongest antimicrobial activity against *E. coli* and *Salm. Typhimurium*; and YD25 exhibited the most potent antimicrobial activity against *S. aureus*.). Since encouraging results have been achieved using mixed LAB, we hypothesize that a mixture of YD6, YD9, and YD25 may be a desirable combination for the prevention and/or treatment of bovine endometritis. Further extensive experiments on the *in vivo* effects of these isolates are warranted.

## Supplemental Information

10.7717/peerj.13177/supp-1 Supplemental Information 1 The characteristic morphologies of colony and cell of isolated LAB.

10.7717/peerj.13177/supp-2 Supplemental Information 2 Amplification results of 16S rRNA gene from five isolates. Lane M: molecular weight marker 2kb; Lane 1:YD6; Lane 2:YD9; Lane 3:YD14; Lane 4:YD18; Lane 5:YD25.

10.7717/peerj.13177/supp-3 Supplemental Information 3 The hemolytic activity results of LAB isolates.

10.7717/peerj.13177/supp-4 Supplemental Information 4 Detection of specific virulence genes in YD6 and YD14. Lane M: molecular weight marker 2kb; Lane : 1-8 Ace Agg Asa1 Cpd CylA ClyB EfaAfs GelE in YD6; Lane: 9-16 Ace Agg Asa1 Cpd CylA ClyB EfaAfs GelE in YD14; Lane :17-24 Ace Agg Asa1 Cpd CylA ClyB EfaAfs GelE negative control.

10.7717/peerj.13177/supp-5 Supplemental Information 5 Raw data for Figure 1.

10.7717/peerj.13177/supp-6 Supplemental Information 6 Raw data for Figures 2-3.

10.7717/peerj.13177/supp-7 Supplemental Information 7 Raw data for Tables 2-4.

## Additional Information and Declarations

### Data Availability

The following information was supplied regarding data availability: The raw measurements are available in the Supplemental Files.
