# Supplementary figures and images for: In vitro evaluation of probiotic properties of lactic acid bacteria isolated from the vagina of yak (Bos grunniens)
Source: PeerJ. 2022 Mar 29;10:e13177. doi: 10.7717/peerj.13177 (PMC8973462; doi:10.7717/peerj.13177)

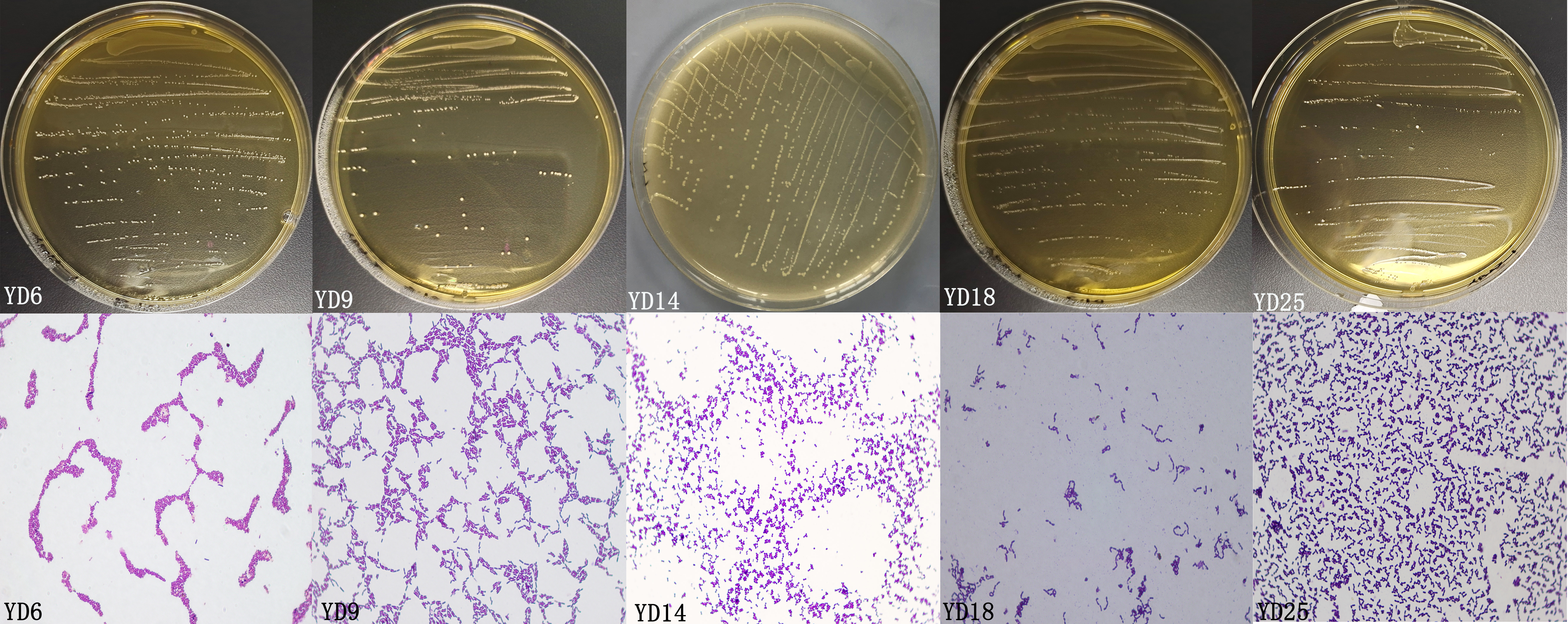

Supplement: Supplemental Information 1 [file peerj-10-13177-s001.png]

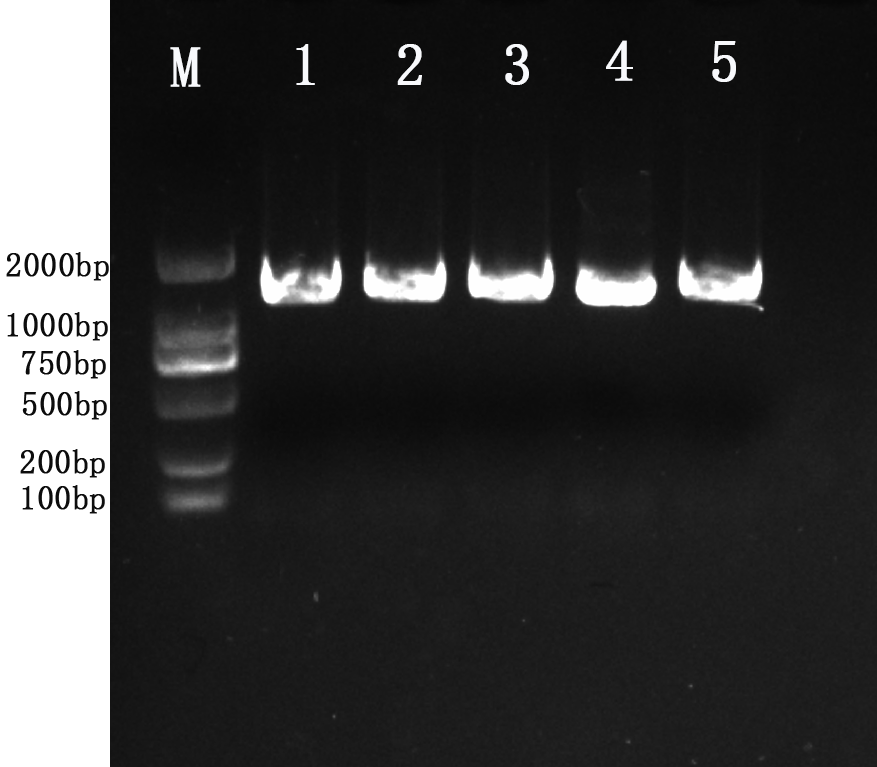

Supplement: Supplemental Information 2 — Lane M: molecular weight marker 2kb; Lane 1:YD6; Lane 2:YD9; Lane 3:YD14; Lane 4:YD18; Lane 5:YD25. [file peerj-10-13177-s002.png]

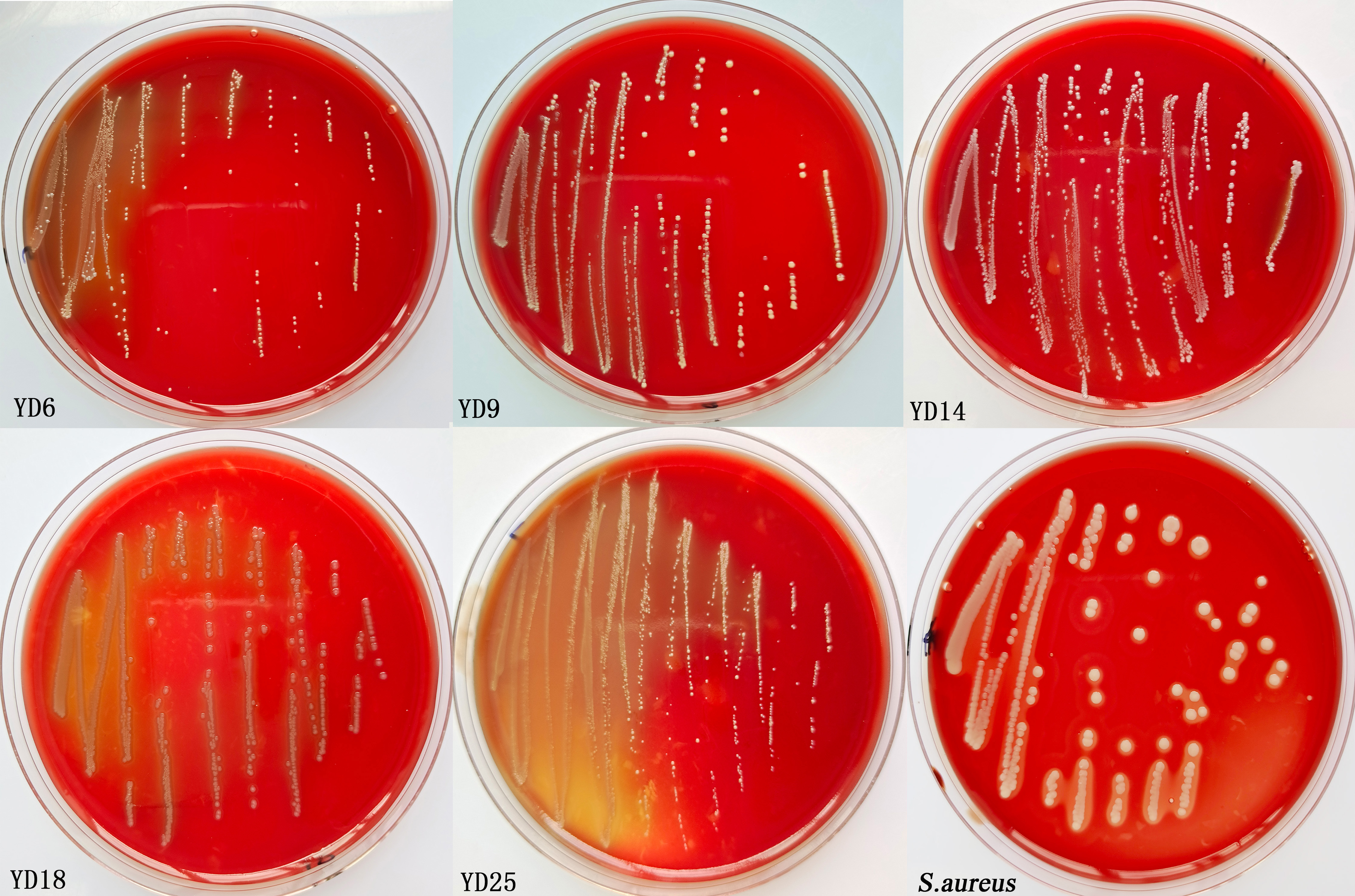

Supplement: Supplemental Information 3 [file peerj-10-13177-s003.png]

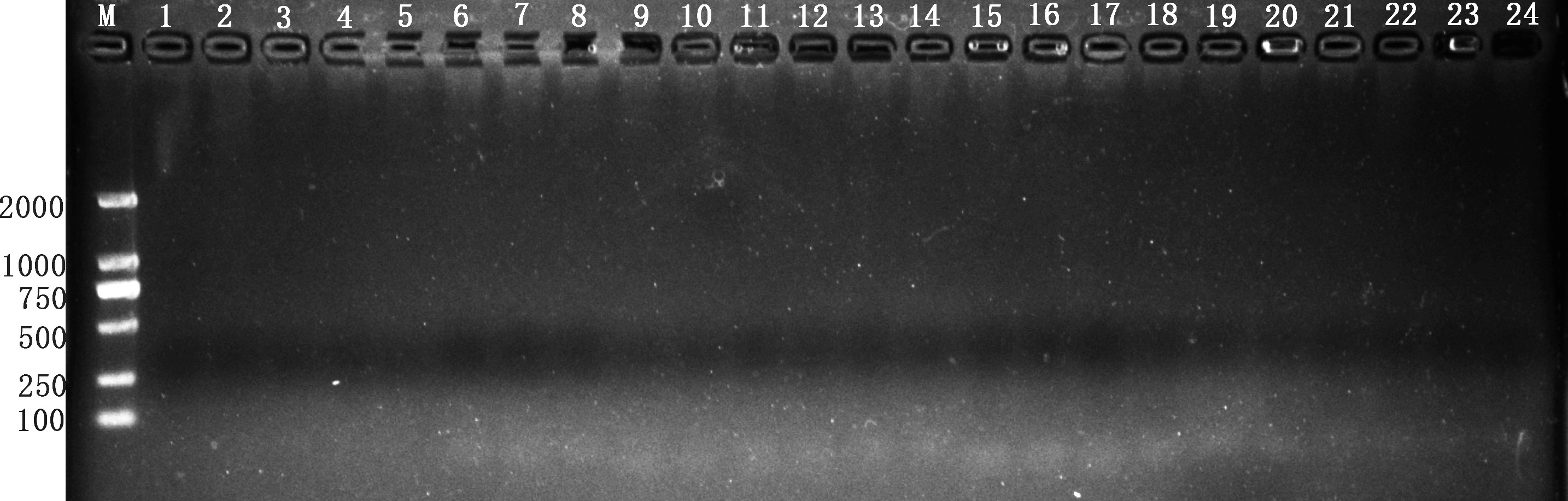

Supplement: Supplemental Information 4 — Lane M: molecular weight marker 2kb; Lane : 1-8 Ace Agg Asa1 Cpd CylA ClyB EfaAfs GelE in YD6; Lane: 9-16 Ace Agg Asa1 Cpd CylA ClyB EfaAfs GelE in YD14; Lane :17-24 Ace Agg Asa1 Cpd CylA ClyB EfaAfs GelE negative control. [file peerj-10-13177-s004.png]
